# Cultural competence of paediatric doctors: A qualitative study in a rural setting

**DOI:** 10.4102/jcmsa.v3i1.204

**Published:** 2025-07-22

**Authors:** Ntandoyenkosi L. Msomi, Suvishka Barath

**Affiliations:** 1 Department of Family Medicine, College of Health Sciences, University of KwaZulu-Natal, Durban, South Africa; 2 Department of Speech Language Therapy, Clinical Support Services, Queen Elizabeth Hospital King’s Lynn, Norfolk, United Kingdom

**Keywords:** rural health, doctors, paediatrics, cultural competence, KwaZulu-Natal

## Abstract

**Background:**

Cultural competence is an important aspect of patient-centred care, particularly in paediatric settings, where doctors from diverse backgrounds interact with families from different cultural contexts. South Africa is a multi-cultural country, with doctors working with people whose concepts of health and healing include traditional beliefs and practices. While the importance of cultural competence is widely acknowledged, limited data exist on how doctors exercise cultural competence in a neonatal intensive care unit (NICU). This study addresses this gap by exploring doctors’ perspectives on providing culturally responsive care in a rural NICU.

**Methods:**

We conducted an exploratory qualitative study, situated within a constructivist paradigm, to explore how doctors understand and practise cultural competence. The constructivist lens guided the exploration of participants’ socially constructed perspectives within their clinical contexts. Nine doctors working in the NICU of a public-sector regional hospital in KwaZulu-Natal Province, South Africa, were purposively sampled. Semi-structured interviews were conducted, and data were analysed thematically.

**Results:**

Five themes were generated: (1) understanding cultural competence, (2) learning cultural competence, (3) importance of clinical settings, (4) professional challenges in displaying cultural competence and (5) evaluating cultural competence. Collectively, these themes highlight the disconnect between the perceived value of cultural competence and the realities of insufficient training, inconsistent application and limited institutional support in high-pressure clinical settings.

**Conclusion:**

While the doctors recognised cultural competence as integral to effective paediatric care, gaps remain in training, implementation and evaluation. Addressing these challenges through structured education may enhance culturally responsive health care delivery.

**Contribution:**

This study contributes to the growing discourse on cultural competence in health care by providing perspectives into its practical application and challenges in a South African paediatric setting.

## Introduction

South Africa is a nation of approximately 63 million people, with a variety of languages, religions and cultural traditions.^1,2^ The country faces increasing service delivery challenges, largely because of a shortage of skilled health care professionals and a lack of resources to provide adequate infrastructure and resources.^3,4^ Many health care facilities, influenced by their geographical locations, continue to reflect racial and ethnic patterns that are rooted in the apartheid era.^5^ In this context, both anecdotal evidence and documented reports reveal instances of racial bias, unequal treatment and language being used as a barrier to accessing health care services.^1,6^

Globally, literature has explored the cross-cultural challenges health care professionals face, focusing on strategies to address cultural differences and enhance cross-cultural communication.^7,8,9,10^ Cultural competency in health care refers to the ability of health care providers to understand, communicate with and effectively interact with patients from diverse cultural backgrounds.^11^ Cultural backgrounds refers not just to race or ethnicity but also includes language, religion, family structure and traditional health beliefs, all of which vary significantly even within the same nationality. It is an essential component for equitable health care delivery, particularly in paediatric settings, where doctors must navigate the complexities of diverse patient backgrounds to provide culturally sensitive and effective care.^12,13,14^ In paediatric health care, cultural competency facilitates improved communication, stronger patient–provider relationships and better health outcomes, by recognising and respecting people’s cultural preferences.^11,15^ Given the increasing cultural diversity within health care settings, cultural competence is recognised as a core skill for health care professionals.^14^ In health care service delivery, the Campinha-Bacote Model is used, which proposes that cultural competence is not a fixed skill but an evolving process requiring health care professionals to actively engage in lifelong learning and self-reflection.^16^

South Africa’s rich cultural diversity presents unique challenges for paediatric doctors. Regional hospitals serve populations with various socio-economic and cultural backgrounds, each with distinct beliefs, traditions and expectations regarding health care. Inadequate cultural competency among doctors may lead to miscommunication, reduced patient satisfaction and disparities in health outcomes.^11,17^ Furthermore, the country’s historical context of racial and socio-economic inequalities underscores the necessity of culturally competent health care to ensure equitable access to quality services.^1,4^

Cultural competency is essential in multi-disciplinary paediatric health care, particularly in neonatal intensive care units (NICUs), where professionals such as paediatricians, neonatologists, nurses, speech and language therapists, lactation consultants and radiographers collaborate to provide patient-centred care.^18^ In these settings, cultural competency facilitates effective communication and collaboration between health care professionals and families from diverse backgrounds. Parents’ cultural beliefs can shape their perspectives of paediatric care, decision-making and adherence to medical recommendations, making cultural competence an essential skill for health care professionals.^19^ A deeper understanding of family-centred and culturally-responsive approaches is essential for ensuring optimal care in paediatric settings, where critical illness, and grandparents involvement add to the complexity of this environment.^19^

Culture is a broad concept that encompasses the knowledge, values, beliefs, assumptions, and practices individuals inherit as members of a specific society or group, or adoptas their circumstances evolve.^9^ Cultural factors shape attitudes towards the health, healing, self-care and health care utilisation, often influencing health outcomes.^20^ Increasing globalisation and migration have also resulted in health care professionals being required to engage with patients from diverse backgrounds, highlighting the need for cultural competence in medical education and practice.^21,22^ Cultural competence therefore needs to extend beyond awareness of different cultural practices to include skills essential for culturally responsive care and the ability to address health disparities linked to socio-cultural factors.^21,23^ It is also closely linked to cultural humility, which involves a lifelong commitment to self-evaluation, recognising personal biases and fostering respectful partnerships with patients, irrespective of their culture.^20^

Despite South Africa’s diversity, research on the cultural competency of paediatric doctors, particularly in KwaZulu-Natal (KZN), is scarce. Given the province’s diverse population and the potential impact of cultural beliefs on health care interactions, understanding doctors’ cultural competency is essential for ensuring optimal patient care and reducing health disparities. Therefore, our central research question guiding our study was: *What is the cultural competency profile of paediatric doctors in KZN, and what is their understanding of how cultural beliefs and practices impact health care delivery*?

## Research methods and design

### Study design

The study adopted a qualitative exploratory research design, positioned within the constructivist paradigm, which emphasises that knowledge is actively constructed by individuals through their engagement with the world and each other.^24^ This paradigm was appropriate for the study’s aim of exploring doctors’ perspectives of cultural competence within the NICU.

### Study setting

The study was conducted in a rural area at one of six regional hospitals in KZN, South Africa, the facility being a referral hospital for two district hospitals and multiple community health care centres. The hospital has a paediatric department, which provides both in- and out-patient services. Doctors in this regional hospital, provide care in wards, outpatient clinics, a kangaroo mother care unit and the NICU.

### Study population

Our study population included medical interns and medical officers working in the NICU. The interns were in the final weeks of their 3-month rotation, having completed 11 months out of their 2-year internship. The medical officers were permanently assigned to the paediatric unit, without rotation to other clinical departments.

### Sampling strategy

Gatekeeper permission was granted by the provincial department of health, the regional hospital’s Chief Executive Officer (CEO) and the paediatric head of the clinical department with the condition that the interviews should not disturb normal operations in the clinical unit. We used purposive sampling of medical practitioners using an emailed invitation letter that was sent by the first author. Participation was dependent on willingness to participate and availability, and limited to the interns and medical officers, irrespective of the amount of time they had worked in the unit. Interviews were conducted in November 2024, in the morning, before the participants began their usual daily routine.

### Data collection

A semi-structured interview guide was employed, which allowed for flexibility in follow-up questions based on the participants’ responses. The interview guide was piloted with two doctors working in the NICU at another hospital in KZN to ensure the data collection tool’s trustworthiness.^25^ All interviews were conducted in English and isiZulu, the official workplace languages. These sessions were held in private spaces, typically in the boardroom, at a time convenient for the participants, each being audio-recorded and lasting 30–45 min. The following questions were asked of all participants:

- What is your understanding of the word ‘culture’?
- How would you define cultural competence?
- How did you learn about cultural competence?
- Do you think cultural competence is essential when working in a paediatric clinical unit? Yes/No and why?
- How would you describe your ability to display cultural competence in your paediatric work duties?
- Are you familiar with any strategies we can use to evaluate cultural competence in your profession?
- What challenges or barriers do you face in providing culturally competent care in the NICU?
- What training or support do you think would help improve cultural competence in your field?

### Positionality and reflexivity

At the time of the study, the first author was employed as a speech and language therapist and lactation consultant at the hospital where the research took place. This role was disclosed to gatekeepers and was known to the study participants. The paediatric discipline has a rigorous training framework for its students and interns. While working at the regional hospital, the first author became interested in exploring how doctors in paediatrics understand cultural competence and its relevance to their discipline.

Reflexivity refers to the researcher’s ability to critically reflect on his role in the knowledge production process.^26^ In this study, the first author remained aware of his positionality throughout data collection, analysis and interpretation. As he conducted interviews with colleagues who worked in the same environment, he needed to ensure that the findings were not influenced by prior familiarity with the setting or participants. To strengthen reflexivity, the first author documented all participant comments, recorded personal reflections during and after interviews, and created reflective summaries immediately following each interview.

While the first author worked within the NICU, the second author was not involved in clinical service delivery at the study site and had no prior relationship with the participants. As an audiologist and doctoral scholar with qualitative research experience, the second author brought an external perspective and expertise. This provided a level of analytical distance that complemented the first author’s insider perspectives. The second author’s role in the data analysis process, including independent coding and theme development, helped to balance subjectivity and enhance the credibility and trustworthiness of the findings.

### Data analysis

Interviews were transcribed verbatim throughout the data collection process, with all identifying information removed to ensure anonymity. The first author reviewed each transcript to identify areas requiring further exploration in subsequent interviews. The transcripts were then imported into NVivo^®^ 10 software (QSR International, 2010) for coding and thematic analysis, guided by established frameworks.^27,28,29^

The first and second authors independently conducted line-by-line open coding of the initial interview. As coding progressed, the authors had ongoing discussions, modifying existing codes and generating new ones. Codes were organised into preliminary themes until data saturation was achieved, at which point no additional coding was necessary. The final themes and sub-themes were confirmed through collaborative review and discussion among the authors. To enhance the readability of quotes used to illustrate the themes, grammatical and typographical errors were corrected. Ellipses were used to indicate omitted text without altering the quote’s meaning, while square brackets provided additional context. Data saturation was reached after interviews had been conducted with nine medical practitioners, when no new themes were derived from the data and no further coding was feasible, according to the established guidance.^30^

### Ethical considerations

Before conducting the study, ethical approval was obtained from the University of KwaZulu-Natal Humanities and Social Sciences Research Ethics Committee (Ref: HSSREC/00007489/2024). The KwaZulu-Natal Provincial Health Research Committee of the Department of Health granted gatekeeper approval (NHRD Ref: KZ_202409_024), and the CEO of the institution provided permission to conduct research on understanding and practice of cultural competence in the NICU. All participants were given written information about the study, and written informed consent was obtained to participate and audio record the interviews. Participation was voluntary, and transcript confidentiality was assured using unique participant identifiers.

## Results

The nine participants included locally and foreign-trained doctors with various cultural backgrounds and experience levels. The diversity of the participants is shown in Table 1.

**TABLE 1:** Participants’ demographics.

Code	Sex	Cultural background	Job title	Years’ of experience	Qualifications	Nationality
P1	F	Zulu	Medical officer	7	MBChB	South African
P2	M	Sunni	Medical intern	< 1	MBBS Foreign	South African
P3	M	Sunni	Medical intern	< 1	MBBS Foreign	South African
P4	F	Multi-cultural	Medical officer	7	MBChB	South African
P5	M	Xhosa	Medical officer	3	MBChB	South African
P6	F	Indian	Medical intern	< 1	MBChB	South African
P7	F	Zulu	Medical intern	< 1	MBChB	South African
P8	M	Zulu	Medical intern	< 1	MBChB	South African
P9	F	Bangeli	Medical officer	24	MBBS Foreign DCH (SA)	Bangladeshi

P, participant; F, female; M, male; SA, South Africa.

The themes and sub-themes that emerged from the doctors’ perspectives of cultural competence and how it relates to their clinical practice are indicated in Table 2.

**TABLE 2:** Themes and sub-themes.

Themes	Sub-themes
1. Understanding of cultural competence	1.1 Definition and scope 1.2 Context of care
2. Learning cultural competence	2.1 Formal education and training 2.2 Practical experience
3. Importance of clinical settings	3.1 NICU-specific relevance 3.2 Family and decision-making dynamics
4. Professional challenges in displaying cultural competence	4.1 Practical challenges 4.2 Maintaining respect
5. Evaluating cultural competence	5.1 Current practices 5.2 Potential improvement

NICU, neonatal intensive care unit.

### Theme 1: Understanding of cultural competence

This theme explores participants’ definitions and interpretations of cultural competence, highlighting the broad scope of the concept. It consists of two sub-themes: definition and scope and context of care.

#### Subtheme 1.1: Definition and scope

Participants defined cultural competence as the ability to understand and respect different cultural backgrounds and beliefs, including recognition of how these factors influence health and well-being:

‘Cultural competence is your understanding of culture, what kind of a person, where they come from, what shapes their ideas, their health, their well-being, their family … so that you can help them in a way that doesn’t contradict what they believe.’ (P5, Male, Medical officer)

‘My understanding is cultural competence is the ability to be able to communicate and interact effectively with people from different cultures.’ (P8, Male, Medical intern)

#### Subtheme 1.2: Context of care

The participants emphasised that cultural competence is essential for understanding the dynamics of caregiving, including the roles of family members and their beliefs:

‘It’s important to find out who is caring for the baby most of the time … being mindful of their beliefs, be it religious, cultural, even national.’ (P2, Male, Medical intern)

‘When we make decisions, especially in our facility, you might have women say: “I need to speak to the father”, because he is the one who makes the final decision.’ (P9, Female, Medical officer)

### Theme 2: Learning cultural competence

This theme highlights how participants acquired cultural competence and underscores the importance of hands-on learning in diverse clinical settings, the two sub-themes being formal education and training, and practical experience.

#### Subtheme 2.1: Formal education and training

The participants noticed that formal instruction on cultural competence was limited and often addressed superficially, such as through ethical discussions:

‘I learned about cultural competence in 6th year [*last year of undergraduate study*]; they told us not to judge patients based on what they are wearing.’ (P6, Female, Medical intern)

‘We looked at cultural competence through ethics during GP [*general practitioner*] training.’ (P8, Male, Medical intern)

#### Subtheme 2.2: Practical experience

Many participants developed cultural competence through on-the-job experience, learning from colleagues, patients and clinical situations:

‘Most of the learning came after working in clinical settings … encountering different cultures and learning from patients themselves.’ (P3, Male, Medical intern)

‘You learn about the cultural effects when working in traditional settings … understanding backgrounds helps integrate practices that don’t alienate patients.’ (P7, Female, Medical intern)

### Theme 3: Importance in clinical settings

The participants noticed that cultural competence is essential in health care, particularly in sensitive environments, the two subthemes being NICU-specific relevance and family-decision making dynamics.

#### Subtheme 3.1: Neonatal intensive care unit-specific relevance

Participants highlighted the unique challenges of cultural competence in NICU settings, where diverse beliefs related to caring for a newborn influence caregiving and medical decisions:

‘In NICU, you have caregivers from different generations with varying cultural beliefs … it’s important to understand these to address their concerns effectively.’ (P2, Male, Medical intern)

‘When breaking bad news, you need to understand how to address families based on their cultural background … it helps when they perceive the issue differently.’ (P8, Male, Medical intern)

#### Subtheme 3.2: Family and decision-making dynamics

This subtheme explores how intra-family cultural differences (such as between maternal and paternal sides) can complicate clinical decision making:

‘In NICU, a baby might belong to two families with conflicting cultural views … this can make decision-making longer and more challenging.’ (P5, Male, Medical officer)

‘Understanding the family hierarchy is essential; for some, decisions are made by the elders, not the mother.’ (P4, Female, Medical officer)

### Theme 4: Professional challenges in displaying cultural competence

This theme relates to the difficulties experienced by the health care teams when dealing with patient cases, with two themes being identified, these being practical challenges and maintaining respect.

#### Subtheme 4.1: Practical challenges

Participants acknowledged the fact that applying cultural competence in real-life scenarios could be complex and emotionally taxing for the medical staff, as their knowledge and experience may put their treatment recommendations in conflict with those of the newborn’s family:

‘Sometimes, patients’ traditional practices conflict with medical advice, like using traditional enemas after discharge … this creates frustration.’ (P6, Female, Medical intern)

‘It’s one thing to understand cultural competence theoretically, but applying it practically is very complicated.’ (P9, Female, Medical officer)

#### Subtheme 4.2: Maintaining respect

Respecting patients’ cultural practices was emphasised as a key aspect of cultural competence:

‘You need to show patients you understand their beliefs … if you judge them, they may not come back, or they’ll return when it’s too late.’ (P4, Female, Medical officer)

‘When you get a translator to help communicate in their language, patients feel more accepted.’ (P3, Male, Medical intern)

### Theme 5: Evaluating cultural competence

This theme relates to how cultural competence is assessed in health care settings and identifies gaps in formal evaluation processes, the two themes being current practices and potential improvements.

#### Subtheme 5.1: Current practices

Participants noted that there are no standardised methods to evaluate cultural competence, with most relying on informal feedback and personal reflection:

‘There’s no formal way to evaluate it [*cultural competence*] … you rely on personal effort to learn from patients and colleagues.’ (P2, Male, Medical intern)

‘We don’t have protocols or tools; it’s often left to individual interest.’ (P7, Female, Medical intern)

#### Subtheme 5.2: Potential improvements

Participants suggested ways to improve the evalution and delivery of culturally competent care, including the incorporation of patient feedback mechanisms and a deeper understanding of patients generational practices and social context:

‘Suggestion boxes for patients to provide feedback on their cultural experiences with doctors could help.’ (P5, Male, Medical officer)

‘Understanding patients’ generational practices and social supports is crucial for effective counselling.’ (P1, Female, Medical officer)

## Discussion

The demographic profile of the doctors who participated in this study reflects that of the South African health care workforce. The findings show a cultural contrast between doctors and their largely homogenous patient population. While the patient community reflects a relatively uniform cultural background, the doctors are culturally diverse, differing in cultural background, nationality, years of experience and training. This emphasises the need for cultural competence training. Without cultural competence upskilling, paediatric care may fail to align with patients’ values and expectations. Therefore, the study’s contribution is timely and important, describing how diverse health care providers can navigate and adapt to the cultural realities of their setting to deliver equitable, respectful paediatric care.

The participants had a variety of responses regarding their understanding of cultural competence and its impact on delivering medical services to their paediatric patients. Regarding the definition and scope, nearly all participants described cultural competence as primarily related to individuals’ or families’ values, belief systems, religion, health and well-being and modes of communication and interaction. Most factors mentioned by doctors when describing cultural competence were consistent with those found in the literature, such as effective cross cultural communication,^11^ the reliance on practical experience rather than formal training^31^ and understanding diverse cultural backgrounds.^32^ These factors play an important role in guiding doctors to provide culturally sensitive and effective health care services to patients and their families. Although doctors who participated mainly work with paediatric populations, they are required to adopt a holistic approach that emphasises the need for assessments and treatments to focus not only on the child but also on the parents, primary caregivers and other family members. By doing so, the input and views of parents and families are considered, complementing the medical services proposed by doctors and ensuring alignment with cultural contexts. This requires doctors to be more attentive and direct when obtaining the child’s background information. However, this can sometimes be challenging because of the lack of formal instruction on how to understand and accommodate South Africa’s diverse cultures, or to address cultural belief systems that might be in conflict with evidence-based best practices.

The participants had limited formal education and training on how to address patients’ families’ unique cultural belief systems within clinical settings and reported having gained most of their experience and knowledge through working with patients and colleagues from diverse cultural backgrounds. Doctors with more work experience tended to have a higher level of skill in resolving issues that arose because of patients’ or families’ cultural backgrounds, this being an invaluable and largely unrecognised resource, and an essential part of instruction for junior staff members. This may be attributed to the fact that limited formal training increases the risk of controversies arising from doctors’ treatment approaches and cultural sensitivity, as cultural belief systems vary significantly across communities, races, religions and families (maternal and paternal).^33^ These findings are consistent with the literature, which suggests that South African doctors are inadequately trained to address challenges stemming from cultural beliefs and practices.^34^ This is particularly evident in contexts where more than 60% of patients have cultural backgrounds and languages that are different from their own.^35^ These findings highlight the need for structured learning using formal classes, case studies and supervised practical implementation.

South Africa has high rates of preterm births, often associated with teenage pregnancy, in both rural and urban areas.^36^ As a result, when mothers are very young, grandparents or other older relatives are often expected to assist in health care decision-making to ensure the infants well-being. In such cases, the mother’s views regarding her infant’s treatment or medical care may be overlooked or influenced by the family. Rabbipal,^37^ notes that preterm infants often require comprehensive management plans to address short- and long-term challenges, as they are at high risk of neuro-developmental difficulties. Doctors are expected to communicate such information carefully to help families make informed and culturally appropriate decisions. This can become a challenge when the infant’s mother desires a treatment plan that differs from the family’s preferences. If such conflicts are not handled sensitively and skilfully by doctors, it may lead to the abandonment of treatment. This emphasises the need for follow-up and monitoring of infants where there is a risk of treatment abandonment due culturally determined disharmony. It also highlights the need to work with families to avoid treatments that compromise the health of a newborn and to encourage use of health care facilities.

Participants highlighted challenges in balancing ‘theoretical knowledge’ gained during medical training with its application in real-life clinical cases. Doctors are expected to provide medical services that are scientifically grounded to ensure objectivity and minimise bias. However, these scientific approaches, which originate from Western medical traditions, often conflict when applied in the South African cultural context, with many families, particularly those from rural areas, preferring traditional medicine over Westernised or modern medicine.^38^ As leaders of the medical team, doctors are tasked with formulating treatment plans that are acceptable to both family members and other multi-disciplinary health care providers involved in the patient’s care. However, accessing written guidance is difficult because of the lack of studies and resources that deal with the impact of culture on health and healing.^39^

There are no standardised cultural competence resources that have been developed specifically for the South African multicultural context that doctors can use in clinical settings. As a result, doctors rely on non-standardised, informal approaches when providing care to patients from diverse cultural backgrounds. They often draw on their own clinical experiences, as well as insights gained from colleagues, patients and communities who share similar belief systems, values and languages. The absence of formal and standardised resources creates a risk of unintended errors in the provision of wholistic care. Doctors recognised the importance of incorporating families into clinical discussions, particularly regarding cultural aspects and belief systems. Families can provide valuable insights to help doctors develop treatment plans that are both unique and culturally acceptable for each case.

### Recommendations

Our findings suggest the need for formal training on cultural competence within medical curricula to properly develop cultural competency in the health care setting. Integrating structured cultural competence training through case studies, role-playing and experiential learning could better prepare doctors to work with patients who have a cultural background different from their own. With many participants learning cultural competence through practical experience rather than formal education, health care institutions should implement ongoing professional development programs that include cultural competence training. Also needed are standardised methods for evaluating cultural competence which could include patient feedback mechanisms, peer evaluations and formal accreditation when meeting acceptable cultural competence standards.

### Limitations and strengths

While qualitative studies emphasise depth over breadth, the small number of participants restricts the generalisability of findings other sites in KZN and South Africa. The study was in a single rural hospital in KZN, which may not fully represent the diverse experiences and challenges doctors face in other regions of the province or South Africa. The study’s strengths included using semi-structured interviews, ensuring that the findings reflect the participants’ authentic perspectives. The study contributes to the growing discourse on cultural competence in medical training and practice, highlighting gaps and opportunities for academic and professional improvements.

## Conclusion

Cultural competence in paediatric practice may be overwhelmed by the nature of the work of saving lives under stressful circumstances. While practical experience serves as a key learning tool for developing cultural competence, formal education and standardised assessment mechanisms require further development. Addressing these gaps through structured training initiatives may enhance health care providers’ ability to deliver culturally competent care, ultimately improving patient outcomes.
